# Study of Cytotoxicity of 3-Azabicyclo[3.1.0]hexanes and Cyclopropa[*a*]pyrrolizidines Spiro-Fused to Acenaphthylene-1(2*H*)-one and Aceanthrylene-1(2*H*)-one Fragments Against Tumor Cell Lines

**DOI:** 10.3390/ijms26083474

**Published:** 2025-04-08

**Authors:** Anton A. Kornev, Stanislav V. Shmakov, Alexandra M. Gryschenko, Yulia A. Pronina, Alexander I. Ponyaev, Alexander V. Stepakov, Vitali M. Boitsov

**Affiliations:** 1 Laboratory of Nanobiotechnologies, Saint-Petersburg National Research Academic University of the Russian Academy of Sciences, Saint Petersburg 194021, Russia; 2 Department of Organic Chemistry, Saint-Petersburg State Institute of Technology, Saint Petersburg 190013, Russia; 3 Department of Chemistry, Saint-Petersburg State University, Saint Petersburg 199034, Russia

**Keywords:** spiro-acenaphthylene-1,2′-cyclopropa[*a*]pyrrolizines, spiro-acenaphthylene-1,2′-bicyclo[3.1.0]hexanes, 3-spiro[3-azabicyclo[3.1.0]-hexanes], cyclopropa[*a*]pyrrolizidines, antiproliferative activity, morphological changes (cytoskeleton), cell motility, tumor cell lines, 1,3-dipolar cycloaddition, azomethine ylides, cyclopropenes

## Abstract

A series of 3-azabicyclo[3.1.0]hexanes and cyclopropa[*a*]pyrrolizidines spiro-fused to acenaphthylene-1(2*H*)-one and aceanthrylene-1(2*H*)-one frameworks have been studied for their in vitro antiproliferative activity against human erythroleukemia (K562), cervical carcinoma (HeLa), melanoma (Sk-mel-2), osteosarcoma (U2OS), as well as murine melanoma (B16) cell lines. Using confocal microscopy, it was found that cultivation with the tested spiro-fused compounds led to the disappearance of stress fibers (granular actin was distributed diffusely in the cytoplasm in up to 56% of treated cells) and decrease in filopodia-like deformations (up to 69% after cultivation), which indirectly suggests a decrease in cell motility. The human melanoma cell line scratch test showed that these cells lose their ability to move after cultivation with the tested spiro-fused compounds and do not fill the scratched strip. This was also supported by docking simulations with actin-related targets (PDB ID: 8DNH, 2Q1N). Using flow cytometry, the impact on the mitochondrial membrane potential showed that the tested compounds led to a significant increase in the number of cells with decreased mitochondrial membrane potential from 10% for the control up to 55–80% for the cyclopropa[*a*]pyrrolizidine adducts. The obtained results support the antitumor effect of the tested spiro-compounds and encourage the extension of the study in order to improve their anticancer activity as well as reduce their toxicological risks.

## 1. Introduction

Cancer is one of the most common and lethal health problem worldwide and is the second leading cause of mortality after cardiovascular diseases. Multiple approaches are applied for cancer treatment, including surgery, chemotherapy, radiation therapy, and immunotherapy. However, the effects of existing therapies are still limited, especially for patients with advanced tumors and metastases. The rise of resistance to current cancer therapy is a significant challenge that can further reduce treatment efficacy and often result in poor outcomes. Therefore, developing effective cancer treatments and finding new medications remain a great scientific challenge [1,2].

Natural products isolated from microorganisms, animals, and plants inspire anticancer drug development, as more than half of the approved anticancer drugs or undergoing clinical trials are natural products or their derivatives [3,4,5,6,7]. The enormous number and structural diversity make them a gift from nature for the discovery of lead molecules. Most often, these substances are complex structural compounds that can be only produced by multistage synthetic procedures. Recent achievements in the synthesis of such complex (poly)heterocyclic products has sparked significant interest in the development of efficient synthetic methods for the synthesis of diverse derivatives and structural analogues of these compounds.

Cycloaddition reactions—since their discovery in late 1920s and introduction to synthetic chemical practice by Otto Diels and Kurt Alder—play a tremendous role in synthetic organic chemistry due to the ease in product formation as well as high regio- and stereoselectivity of these processes. Among them, reactions of 1,3-dipolar cycloaddition have long been recognized as important for creating a variety of five-membered carbo- and heterocycles. Azomethine ylides—1,3-dipoles of allyl-type—are universal building blocks widely used in organic synthesis. The ability of azomethine ylides to be involved in the cycloaddition reactions with a variety of dipolarophiles has attracted enhanced attention. Using new stable or in situ generated building blocks allows their further modification and diversification. Azomethine ylide cycloadditions to (cyclo)alkenes are typical reactions that allows for the construction of a variety of (poly)heterocyclic scaffolds (including azabicyclo[3.1.0]hexanes and azabicyclo[3.3.0]octanes) [8,9,10,11,12].

Fused nitrogen-containing heterocycles with a 1-azabicyclo[3.3.0]octane moiety (such as pyrrolizines and pyrrolizidine alkaloids) are common structural components of natural products, as well as their analogues. They are challenging synthetic targets with a broad spectrum of activity (such as antimicrobial [13], antitumor [14,15,16,17], anti-inflammatory [18,19,20], anticoagulant [21]) that are of interest to many medicinal chemists [22,23,24].

Azabicyclo[3.1.0]hexane scaffolds represent another important fused nitrogen-containing heterocyclic structural component that has been found in many natural products [25,26], which are part of pharmaceutical preparations [27,28,29,30] or key synthetic intermediates [31,32,33]. Their broad spectrum of activity includes anti-inflammatory [20], antitumor [34], anti-neurodegenerative [35], antibacterial [36,37], and antiviral (against SARS-CoV-2) activity [38], as well as their use as anti-addiction medication (antagonists of opioid receptors [28,33] and dopamine D3 receptor [39]).

Acenaphthylene (cyclopenta[*de*]naphthalene) frameworks are an important scaffold found in many natural products (Artemisia capillaris, Tuber canaliculatum, Tuber borchii, rhizomes of Musa basjoo) and therefore could serve as an inspiration for the development of new therapeutics [40,41,42,43]).

**Figure 1:**
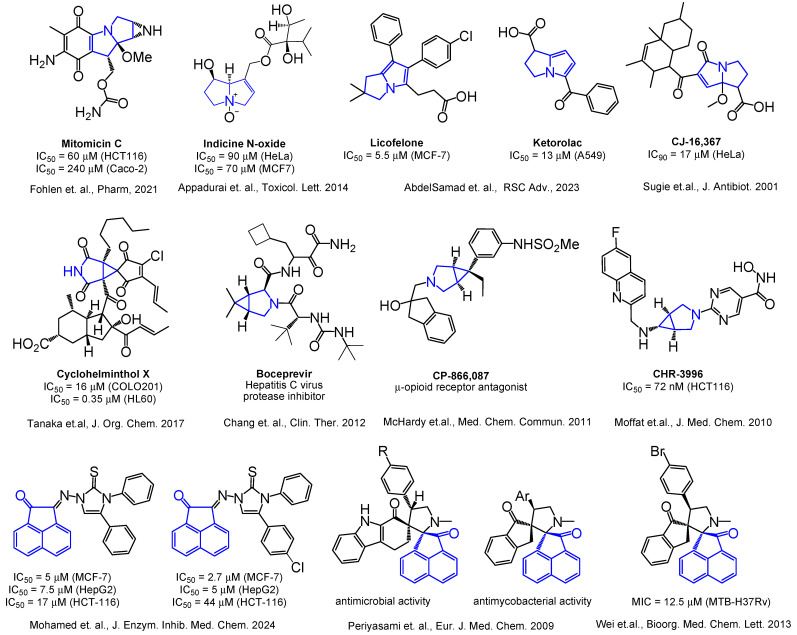
Selected examples of biologically active acenaphthylenes, azabicyclo[3.1.0]hexanes, and 1-azabicyclo[3.3.0]octanes (pyrrolizines or pyrrolizidines) [14,15,16,17,25,27,29,34,41,42,43].

Selected examples of biologically active acenaphthylenes, azabicyclo[3.1.0]hexanes, and 1-azabicyclo[3.3.0]octanes (pyrrolizines or pyrrolizidines) are presented in Figure 1.

In previous studies by our research group, differently substituted cyclopropenes were widely used as dipolarophiles in 1,3-dipolar cycloaddition reactions with azomethine ylides generated from corresponding carbonyl derivatives and *α*-amino acids. Derivatives of 11*H*-indeno[1,2-*b*]quinoxalin-11-one [44], tryptanthrin [45], isatin [46,47], alloxan [48], and ninhydrin [49,50] were used as the ketone components. The products of these reactions were pharmacologically interesting spiro-fused 1-azabicyclo[3.3.0]octanes and 3-azabicyclo[3.1.0]hexanes, some of which were identified as exhibiting in vitro antiproliferative activity [51,52,53].

We here report the study of 3-azabicyclo[3.1.0]hexanes and cyclopropa[*a*]pyrrolizidines spiro-fused to an acenaphthylene-1(2*H*)-one and aceanthrylene-1(2*H*)-one framework (readily available via one-pot three-component 1,3-dipolar cycloadditions of various cyclopropenes with azomethine ylides generated in situ from corresponding 1,2-dicarbonyl compound) for their antiproliferative activity against selected tumor cell lines as well as for their morphological changes, cell motility, and mitochondrial membrane potential changes under treatment with the most active products.

## 2. Results and Discussion

### 2.1. Chemistry

**Figure 2:**
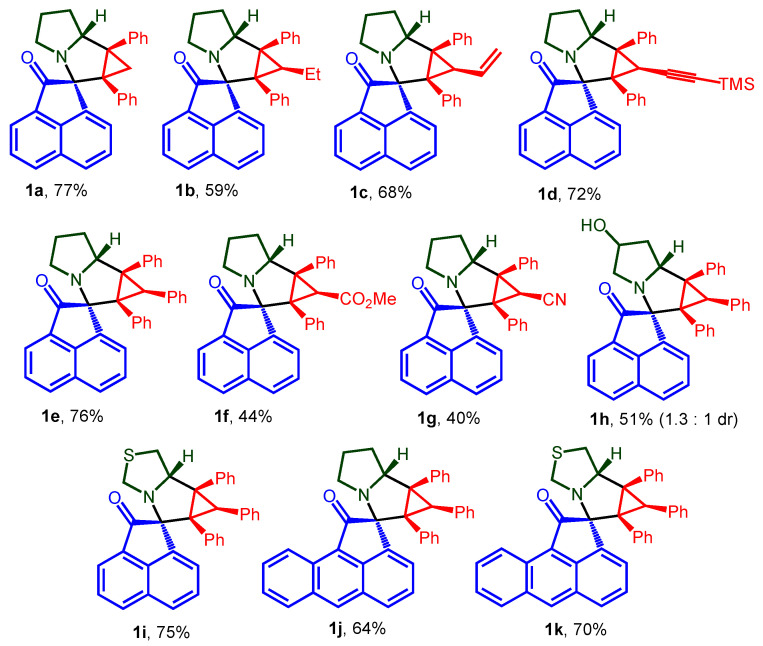
Structures of acenaphthylene-1,2′-cyclopropa[*a*]pyrrolizines and their aceanthrylene analogues **1a**–**k**.

**Figure 3:**
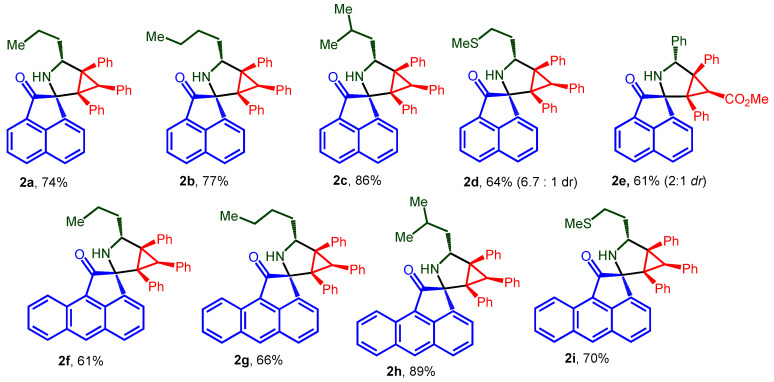
Structures of acenaphthylene-1,2′-bicyclo[3.1.0]hexanes and their aceanthrylene analogues **2a**–**i**.

The desired spiro-fused acenaphthylene-1,2′-cyclopropa[*a*]pyrrolizines and acenaphthylene-1,2′-bicyclo[3.1.0]hexanes, as well as their aceanthrylene analogues, were synthesized by our previously developed methodology using one-pot three-component 1,3-dipolar cycloaddition reactions of substituted cyclopropenes with in situ generated acenaphthylene- or acenaphthylene-derived azomethine ylides with an overall isolation yield up to 89%. The structures used in this study, racemic spiro-adducts **1a**–**k** and **2a**–**i**, are shown in Figure 2 and Figure 3, while the scheme of their synthesis is given in SI (Scheme S1). Structures of cycloadducts were assigned on the basis of NMR spectra analysis and unequivocally verified by X-ray crystal analysis (synthesis and structure elucidation was described in detail by us earlier [47,54]).

An in silico analysis was performed to preliminarily determine whether the synthesized spiro-fused acenaphthylene-1,2′-cyclopropa[*a*]pyrrolizines and acenaphthylene-1,2′-bicyclo[3.1.0]hexanes as well as their aceanthrylene analogues have drug-like properties; their physicochemical profile was determined using the free online software SwissADME (version 3.0, http://www.swissadme.ch/ accessed on 5 February 2025). The molecular descriptors were calculated according to Veber’s rule and Lipinski’s rule of five. According to Veber’s rule, orally active drugs should not violate the following criteria: number of hydrogen bond acceptor < 10 and topological polar surface area < 140 Å^2^. According to Lipinski’s rule, orally active drugs should not violate more than one of the following criteria: molecular weight < 500 Da, number of rotatable bonds < 10, number of hydrogen bond acceptors < 10, number of hydrogen bond donors < 5, octanol/water partition coefficient < 5, and topological polar surface area < 140 Å^2^. The obtained results are presented in Table 1.

Since many drugs used to treat cancer are toxic and have a variety of side effects, it is crucial during new substance development to pay attention to their ADMET properties, with an emphasis on toxicity. The pharmacokinetic parameters that involve absorption, distribution, metabolism, excretion, and toxicity (ADMET) are considered the main causes of failure in developing drugs derived from natural or synthetic products. Here, ADME studies and toxicity profiling were estimated in silico using the online website “https://preadmet.webservice.bmdrc.org/ (5 February 2025)”. Human intestinal absorption (HIA), the blood–brain barrier (BBB), in vitro plasma protein binding (PPB), and the solubility and inhibition of CYP2D6 were selected as ADME descriptors. Carcinogenicity (rat and mouse), mutagenicity (according to Ames test), and in vitro hERG inhibition (cardiotoxicity) were selected as toxicity descriptors. The obtained results are presented in Table 2. As can be seen from the table, the obtained results suggest that the compounds have a good intestinal absorption and plasma protein binding; however, the compounds have a low solubility and low permeation potential in the brain with regard to bioavailability in the CNS.

### 2.2. Biology

**Figure 4:**
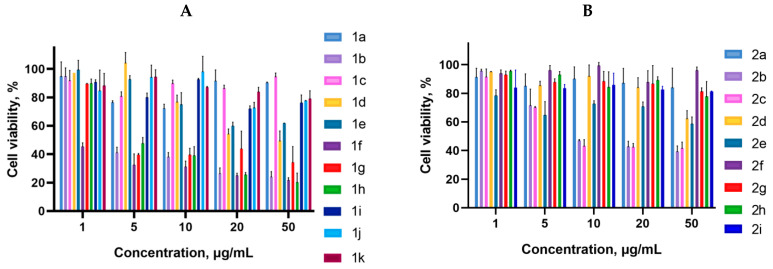
Cytotoxicity of racemic spiro-fused cyclopropa[*a*]pyrrolizines **1a**–**k** (**A**) and 3-azabicyclo[3.1.0]hexanes **2a**–**I** (**B**) against the K562 cell line for 72 h.

**Figure 5:**
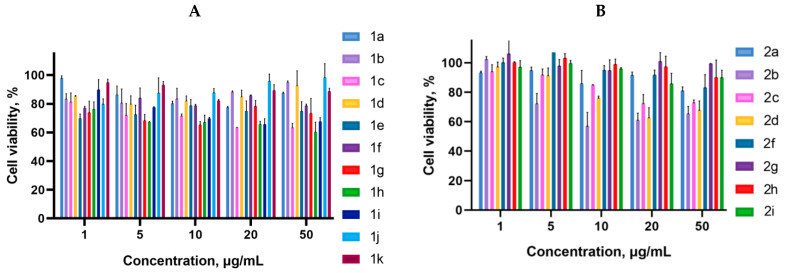
Cytotoxicity of racemic spiro-fused cyclopropa[*a*]pyrrolizines **1a**–**k** (**A**) and 3-azabicyclo[3.1.0]hexanes **2a**–**i** (**B**) against the HeLa cell line for 72 h.

**Figure 6:**
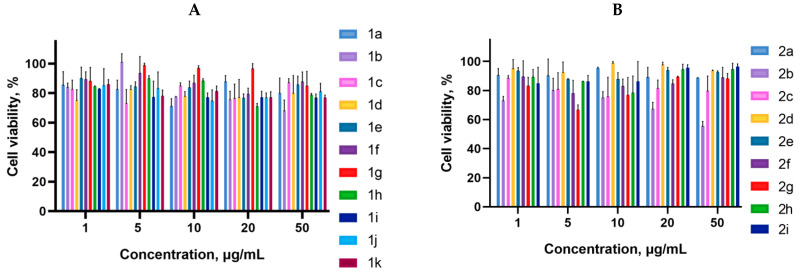
Cytotoxicity of selected racemic spiro-fused cyclopropa[*a*]pyrrolizines **1a**–**k** (**A**) and 3-azabicyclo[3.1.0]hexanes **2a**–**i** (**B**) against the Sk-mel-2 cell line for 72 h.

**Figure 7:**
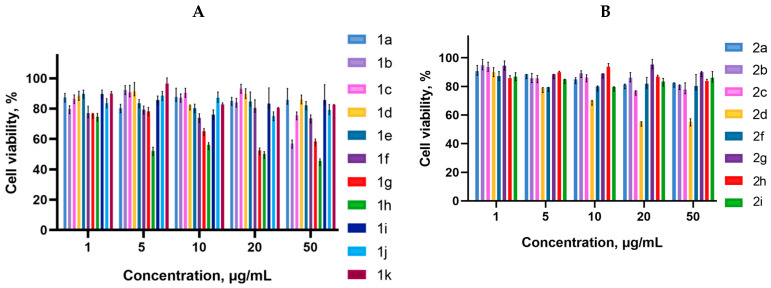
Cytotoxicity of racemic spiro-fused cyclopropa[*a*]pyrrolizines **1a**–**k** (**A**) and 3-azabicyclo[3.1.0]hexanes **2a**–**i** (**B**) against the U2OS cell line for 72 h.

**Figure 8:**
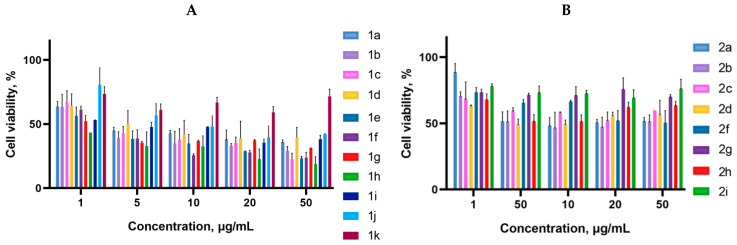
Cytotoxicity of selected racemic spiro-fused cyclopropa[*a*]pyrrolizines **1a**–**k** (**A**) and 3-azabicyclo [3.1.0]hexanes **2a**–**i** (**B**) against the B16 cell line for 72 h.

Cancer cells are valuable in vitro model systems that are widely used in cancer research and drug discovery. Their use is mainly linked to their possibility to provide for experimental purposes an unlimited source of biological material. Here, the in vitro MTS assay was used to determine the antiproliferative activity of synthesized 3-azabicyclo[3.1.0]hexanes and cyclopropa[*a*]pyrrolizidines spiro-fused to acenaphthylene-1(2*H*)-one and aceanthrylene-1(2*H*)-one frameworks against human erythroleukemia (K562), cervical carcinoma (HeLa), melanoma (Sk-mel-2), osteosarcoma (U2OS), as well as murine melanoma (B16) cell lines. It was found that the synthesized spiro-fused derivatives significantly reduced the cell proliferation in a time- and concentration-dependent manner. The results of these investigations for 72 h are presented in Figure 4, Figure 5, Figure 6, Figure 7 and Figure 8. IC_50_ values of the most active spiro-fused adducts against the tested cell lines for 72 h are presented in Table 3.

It is obvious from the obtained data that spiro-fused[cyclopropa[*a*]pyrrolizidine products usually show better antiproliferative activity as compared to their spiro-fused 3-azabicyclo[3.1.0]hexane analogues. Adducts spiro-fused to acenaphthylene-1(2*H*)-one are usually more active compared to those with aceanthrylene-1(2*H*)-one frameworks.

Indeed, among the spiro-fused 3-azabicyclo[3.1.0]hexanes, only adducts **2b** and **2c** exhibited significant antiproliferative effects, with a half maximal inhibitory concentration (IC_50_) around 14 ± 4 μg/mL (against K562 cell line), which equals to 25–27 μM.

Human erythroleukemia (K562) and murine melanoma (B16) were the most sensitive to the screened spiro-fused products among the tested cancer cell lines with IC_50_ ranging from 2 ± 1 to 11 ± 5 μg/mL (72 h), which equals to 4–20 μM.

It was noticed that among the spiro-fused [cyclopropa[*a*]pyrrolizidines, adduct **1f** with a methoxycarbonyl-substituted cyclopropane ring shows better antiproliferative effects, with an IC_50_ value of 2 ± 1 μg/mL (which equals to 4 μM) against the K562 cell line, while ethyl (**1b**), cyano (**1g**), and phenyl (**1h**)-substituted spiro-adducts show antiproliferative effects with an IC_50_ value of around 6 ± 2 μg/mL (which equals to 12–17 μM).

At the same time, all the tested spiro-fused [cyclopropa[*a*]pyrrolizidines show antiproliferative effects against the B16 cell line (except the adduct spiro-fused to the aceanthrylene-1(2*H*)-one framework **1k**) with IC_50_ values ranging from less than 2 ± 1 to 11 ± 5 μg/mL, which equals to 4–20 μM.

Based on obtained data, compounds that have shown better antiproliferative activity were selected for further evaluations of their impact on cytoskeletal morphology, cell motility, and mitochondrial membrane potential.

### 2.3. Actine Cytoskeleton Changes

The actin cytoskeleton is the primary force-generating machinery in the cell, and it is a critical component in a broad diversity of cellular events, such as cell differentiation and cell motility, as well as cell proliferation and cell death regulation [55,56,57,58,59,60]. The ability of the actin cytoskeleton to participate in various cellular processes mainly depends on its dynamic restructuring, which is constantly occurring in response to cellular changes. The reorganization of the actin cytoskeleton caused by tumor transformation leads to changes in cell motility [61,62,63,64]. The structural features of actin organization can serve as criteria for assessing the metastasis potential [65].

The structure of the actin cytoskeleton of HeLa, Sk-mel-2, and B16 cells was assessed after the cultivation with 3-azabicyclo[3.1.0]hexanes and cyclopropa[*a*]pyrrolizidines spiro-fused to acenaphthylene-1(2*H*)-one and aceanthrylene-1(2*H*)-one moieties by the presence of filopodia-like protrusions and the availability of stress fibers.

**Figure 9:**
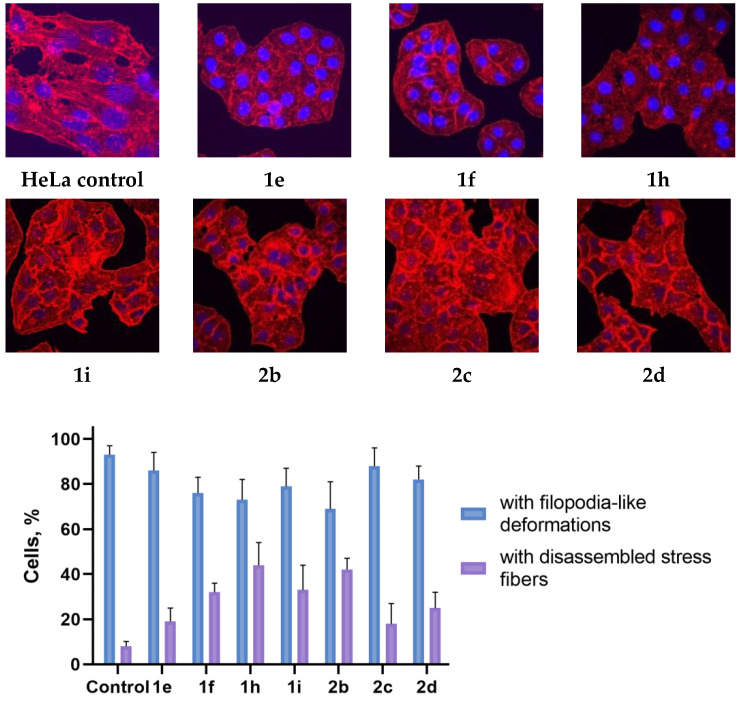
Microscopic images of treated cells and state of actin cytoskeleton of HeLa cells after cultivation with compounds **1e**, **1f**, **1h**, **1i**, **2b**, **2c**, and **2d** (10 μg/mL).

**Figure 10:**
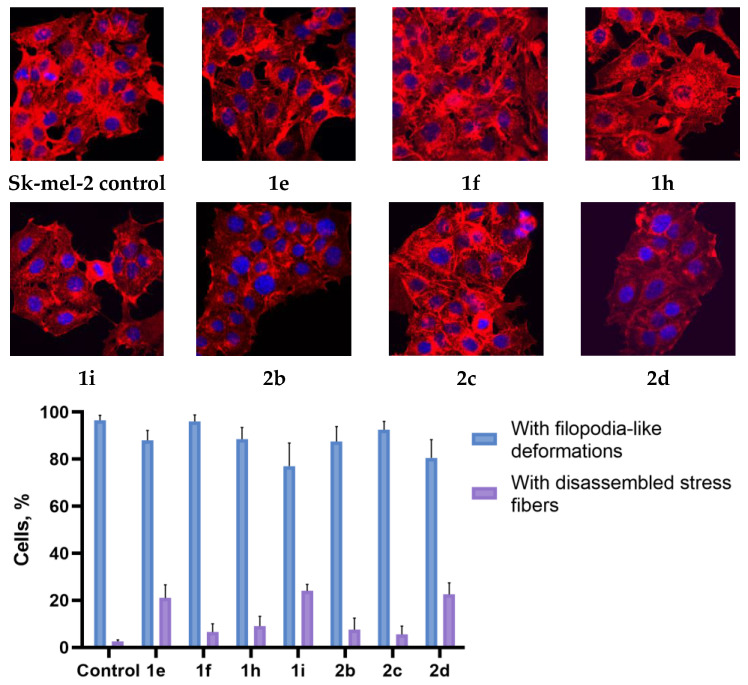
Microscopic images of treated cells and state of actin cytoskeleton of Sk-mel-2 cells after cultivation with compounds **1e**, **1f**, **1h**, **1i**, **2b**, **2c**, and **2d** (10 μg/mL).

**Figure 11:**
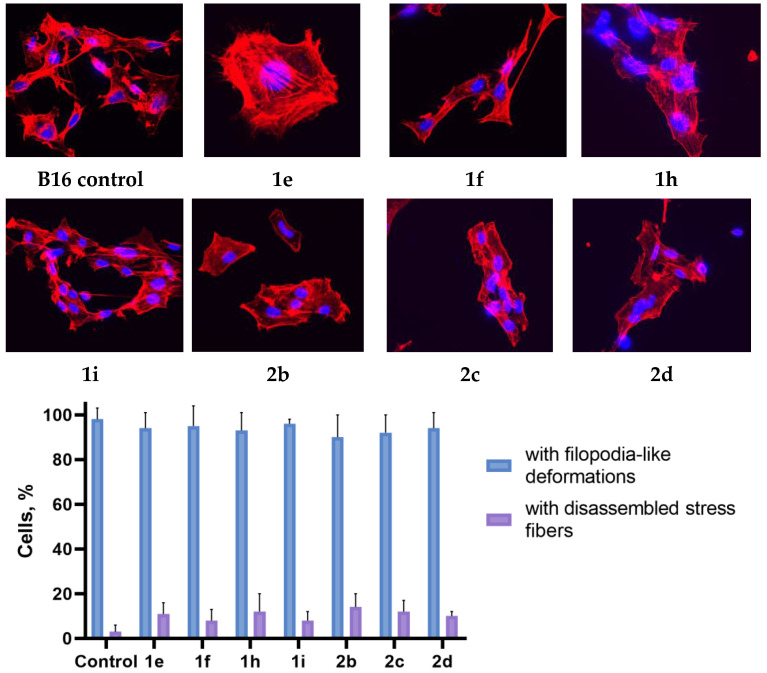
Microscopic images of treated cells and state of actin cytoskeleton of B16 cells after cultivation with compounds **1e**, **1f**, **1h**, **1i**, **2b**, **2c**, and **2d** (10 μg/mL).

Using confocal microscopy, it was found that cultivation with the studied spiro-fused cyclopropa[a]pyrrolizidines **1e**, **1f**, **1h**, **1i**, and 3-azabicyclo [3.1.0]hexanes **2b**, **2c**, and **2d** led to significant changes in the actin cytoskeleton structure of HeLa cells, leading to the disappearance of stress fibers (granular actin was distributed diffusely in the cytoplasm in up to 56% of treated cells) and changes in the number of filopodia-like deformations (reduced up to 69% after cultivation). Such changes in the cytoskeleton may indicate a change in the motor activity of cells, which may indicate a decrease in the ability of tumor cells to metastasize. At the same time, experimental effects did not cause the fragmentation of the nucleus, which indicates the absence of pro-apoptotic activity. A similar (though less expressed) effect on the state of the actin cytoskeleton after cultivation with the studied compounds is also manifested on cells of the Sk-mel-2 and B16 cell lines. Data on actin cytoskeleton structure as well as pie charts demonstrating the percentage of cells with filopodia-like deformations and disassembled stress fibers are combined in Figure 9, Figure 10 and Figure 11.

### 2.4. Inhibition of Cell Motility Evaluated by Scratch-Test

One of the most basic and ancient cellular behaviors is cell motility that is caused by cell invasion and metastasis. Cancer metastases—the cancer cells spread from the primary site to distant organs—continue to be a significant clinical hurdle in cancer diagnosis and treatment. The main problem in understanding the spread of metastatic tumors is that this process cannot be directly observed or manipulated. The inhibition of cell migration was associated with massive morphological changes and the reorganization of the actin cytoskeleton. The scratch test is a simple method for assessing the effects of various influences on cell motility and metastasis.

**Figure 12:**
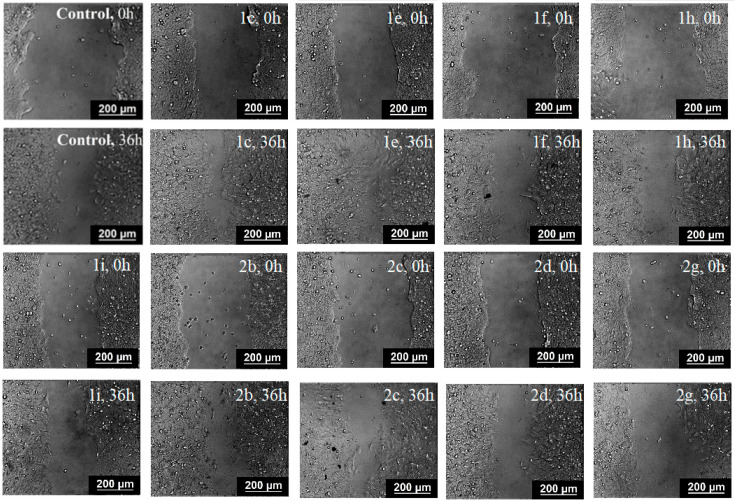

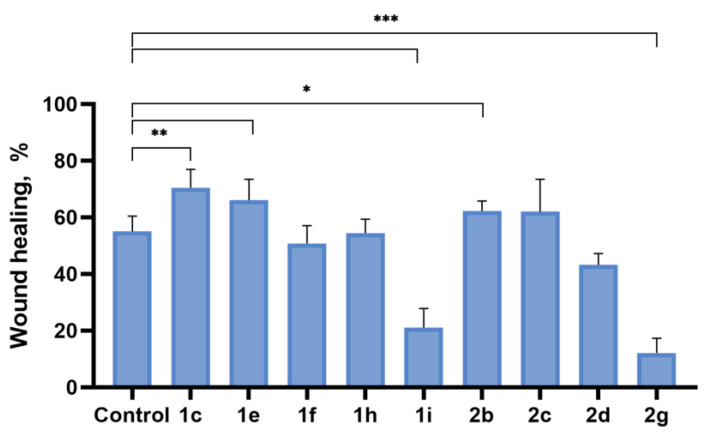
Microscopic images of the Sk-mel-2 cell wound area in the scratch assay and wound area (%) in the scratch assay after 36 h incubation with spiro-fused cyclopropa[*a*]pyrrolizidines **1c**, **1e**, **1f**, **1h**, **1i**, and 3-azabicyclo[3.1.0]hexanes **2b**, **2c**, **2d**, and **2g**. *p* value < 0.05 (*), 0.01 (**), 0.001 (***).

To assess the potential ability of spiro-fused cyclopropa[*a*]pyrrolizidines **1c**, **1e**, **1f**, **1h**, **1i**, and 3-azabicyclo [3.1.0]hexanes **2b**, **2c**, **2d**, and **2g** to inhibit metastasis associated with cell motility, a scratch test was performed on the human melanoma cell line (Sk-mel-2). Different fields were analyzed by a bright field: for fast and non-toxic cell visualization, each scratch area was photographed at 0 and 36 h. The result is shown in Figure 12. Nontreated Sk-mel-2 cells filled the scratched strip at 55 ± 5%, while under treatment with spiro-fused cyclopropa[*a*]pyrrolizidines **1c**, **1e**, **1f**, **1h**, **1i**, and 3-azabicyclo[3.1.0]hexanes **2b**, **2c**, **2d**, and **2g**, cells filled 70 ± 6, 66 ± 7, 50 ± 6, 54.0 ± 5, 21 ± 7, 62 ± 4, 62 ± 4, 43 ± 4, and 12 ± 5% of the scratched strip, respectively. Therefore, the treated Sk-mel-2 cells lose their ability to move and do not fill the scratched strip; however, the structure activity relationship needs further evaluation. The presented results indicate that the tested compounds can block the cellular movement of tumor cells.

### 2.5. Mitochondrial Membrane Potential (∆Ψm) Changes

Mitochondria play a crucial role in regulating cell survival and inducing apoptotic cell death. One of the key indicators of mitochondrial activity is the mitochondrial membrane potential. The loss of mitochondrial membrane potential due to mitochondrial dysfunction can lead to apoptosis.

Apoptosis—a programmed cell death process—plays an important role in the development and progression of malignant tumors. Apoptosis can be initiated by external (extracellular) or intracellular factors. For example, it can result from hypoxia, hyperoxia, subnecrotic damage by chemical or physical agents, cross-binding of the corresponding receptors, disruption of cell cycle signals, removal of growth and metabolic factors, etc. Despite the variety of initiating factors, there are two main ways of signaling apoptosis: a receptor-dependent (external) signaling pathway involving cell death receptors and a mitochondrial (intrinsic) pathway.

The key event of the mitochondrial apoptosis pathway is an increase in the permeability of the outer mitochondrial membrane, leading to a decrease in membrane potential and high-amplitude swelling of mitochondria due to osmotic imbalance. A decrease in the mitochondrial membrane potential is a landmark event of early apoptosis.

**Figure 13:**
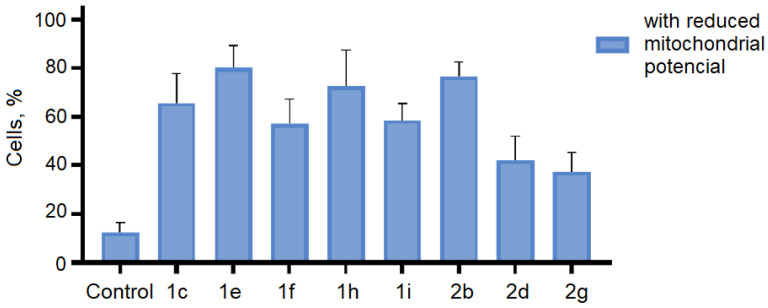
Changes in the mitochondrial membrane potential (∆Ψm) of **K562** cells treated with the studied compounds.

To evaluate functional changes in the mitochondria of K562 cells treated with the studied compounds containing cyclopropane[*a*]pyrrolysine or 3-azabicyclo[3.1.0]hexane fragments, we measured the fluorescence intensity of the JC-1 dye, which depends on changes in the mitochondrial membrane potential (∆Ψm). Healthy cells with a high membrane potential accumulate JC-1 aggregates, which leads to red fluorescence, while damaged cells with low membrane potential exhibit green fluorescence (the higher the JC-1 fluorescence ratio values, the higher the damage to the cells). The research results are shown in Figure 13.

It was found that cultivation with the studied spiro-fused cyclopropa[*a*]pyrrolizidines 1c, 1e, 1f, 1h, 1i, and 3-azabicyclo[3.1.0]hexanes 2b, 2d, and 2g at a concentration of 10 μg/mL led to significant changes in the mitochondrial membrane potential of K562 cells. The number of cells with a decreased mitochondrial membrane potential was increased from 10% for the control, up to 55–80% of cells for cyclopropa[*a*]pyrrolizidine adducts, and up to 35–75% for azabicyclo[3.1.0]hexane adducts. The observed decrease in the mitochondrial membrane potential may be a landmark event of early apoptosis.

### 2.6. Molecular Docking

To support the observed results, docking simulations of spiro-fused cyclopropa[*a*]pyrrolizidines 1b,1c, 1e, 1f, 1g, 1i, and 3-azabicyclo[3.1.0]hexanes 2b, 2c, and 2d were performed with the most abundant, highly conserved main structural protein in the cells—actin. There are two isoforms of non-muscle actin in the cytoplasm of mammalian cells (**β**- and **γ**-actin), which are essential for cell survival. They differ by only four amino acids at the N terminus (positions 1, 2, 3, and 9) [66]. Since the original crystal structure determination of G-actin in the complex with DNase I [67], lots of actin structures have been reported. The majority of them have been obtained as complexes with small molecules and actin-binding proteins. It was noticed that the actin monomer conformation is mainly the same, irrespective of the bound molecule or nucleotide state. The cryo-electron microscopy structure of the non-muscle **β**-actin (8DNH, 2.99 Å) [68] and the X-ray structure of the actin dimer cross-linked between residues 41 and 374 (2Q1N, 2.70 Å) [69] were used for this study. Docking to both known clefts (DNase I-binding or nucleotide and target-binding or hydrophobic) were performed.

**Figure 14:**
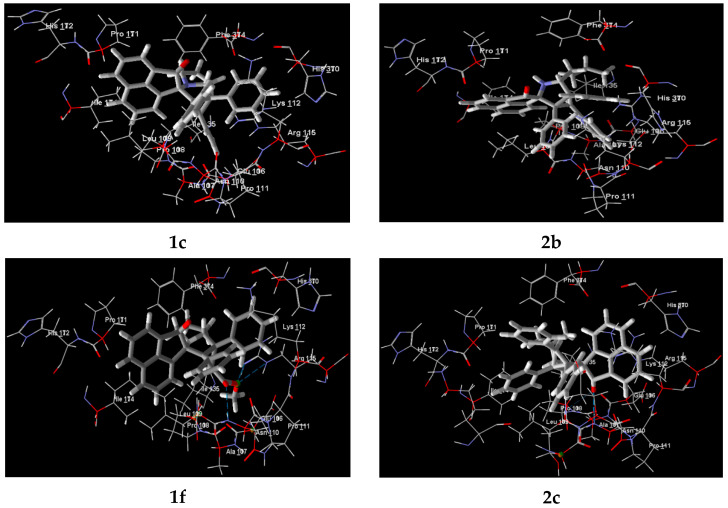
Docked view of **1c**, **1f**, **2b**, and **2c** with the target protein (PDB ID: 8DNH).

The protein structures were obtained from a protein data bank and prepared for a docking study by Molegro Virtual Docker 6.0. The docking results were examined using the pose organizer and the ligand energy inspector tool, the results were tabulated, and the docked view was extracted. The results of docking studies are shown in Table 4 and Table S1 and Figure 14 and Figure S6. According to obtained results, the affinity for the target-binding (hydrophobic) cleft of actin targets was always bigger compared to the nucleotide cleft, with the Rerank Score ranging from −90 to −115 and from −22 to −99 arbitrary units, correspondingly (Table S1, entries 1–9). The predicted binding models also showed that adducts (while fitting within the cleft) arranged usually so that for 8DNH carbonyl oxygen of acenaphthylene moiety directed out of cleft (for 2b to Leu 109, Pro 171, His 172, Ile 174 and cyclopropane ring directed to Asn 110, Pro 111, Lys 112, His 370), while for 2Q1N it directed inside the cleft (for 2b to Val 139, Tyr 143 and cyclopropane ring to Leu 110, Lys 113, Arg 116) (Figure S5). Such an obtained result is consistent with the data on actine cytoskeleton changes and the inhibition of cell motility, and can be explained by an imbalance in actin polymerization and depolymerization processes that are constantly occurring in the cell.

It is known that most of the clinically used anticancer drugs induce apoptosis through genotoxic stress at various stages of the cell cycle and the activation of p53. The p53 protein (known as the guardian of the human genome and acting as a tumor suppressor) plays a vital role in preventing tumor development. This function of p53 is antagonized by its negative regulator protein MDM2 (key oncogenic protein) via multiple mechanisms. The inhibition of the MDM2-p53 interaction may be effective in treating cancer [70,71]. Currently, nutlin, spirooxindole, isoquilinone, and piperidinone derivatives that act as such inhibitors are found to be promising in the treatment of cancer. In order to check whether new developed adducts can be such inhibitors, docking simulations were performed with recent X-ray structures of MDM2 proteins with small molecule inhibitors (PDB ID 7BJ6, 1.59 Å and 7BIR, 2.02 Å) [72]

**Figure 15:**
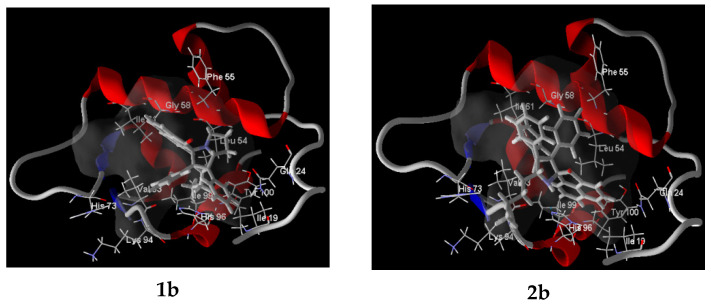
Predicted binding models of compounds **1b** and **2b** within the target-binding cleft of MDM2 protein (for PDB ID: 7BJ6).

The results of docking studies are shown in Table 5 and Figure 15 and Figure S7. According to the obtained results, the affinity of adducts towards the MDM2 protein were always slightly smaller compared to the structure’s own (“active”) ligand (Table 5), while 7BJ6’s active ligand TVK showed comparable results to the adducts when placed at 7BIR (Table 5, entries 1 and 22). The predicted binding models also showed that adducts, while fitting within the cleft, arranged usually so that for 7BJ6 acenaphthylene moiety located in Leu pocket for **2b**, **2c**, **2d** (with carbonyl oxygen directed out of cleft) and in Trp pocket for **1b**, **1c**, **1e**, **1f**, **1g**, **1i**.

Such an obtained result is consistent with the data obtained through the antiproliferative activity study and allows for speculation about one of the possible mechanisms of action, while further structure modification is needed in order to find the best-fitted adduct. Still, the obtained result indicates the potencies of the newly developed spiro-fused adducts as possible antitumor agents.

## 3. Materials and Methods

### 3.1. Chemistry

^1^H (400 MHz) and ^13^C (101 MHz) NMR spectra were recorded with a Bruker Avance 400 spectrometer. Chemical shifts are reported in ppm relative to solvent residual signals (7.26 and 77.16 ppm for ^1^H and ^13^C in CHCl_3_; 2.50 and 39.52 ppm ^1^H and ^13^C in DMSO-*d*_5_) as internal standards. Melting points were determined using a Boetius instrument. Cyclopropenes were prepared according to the literature data [73,74,75,76,77], while α-amino acids**,** acenaphthylene-1,2-dione, and aceantrylene-1,2-dione were obtained from commercial sources. The reaction course, purity, and individuality of the compounds were monitored by TLC on Silufol UV-254 plates. Preparative TLC was performed on a 5–40 mesh silica gel, eluting with a petroleum ether–ethyl acetate mixture.

The general procedure for the synthesis of racemic spiro adducts **1a**–**k** and **2a**–**i** is as follows.

A mixture of the corresponding α-dicarbonyl compound (0.3 mmol), cyclopropene (0.3 mmol), and α-amino acid (0.6 mmol) was stirred at 60 °C in MeOH (8 mL) for 12 h. After completion of the reaction as monitored by TLC, the solvent was removed under reduced pressure. The residue was subjected to silica gel PTLC using a mixture of hexane-ethyl acetate as an eluent to obtain the desired spiro-fused cycloadducts. Spectral and physical data for all the obtained products were identical to those described by us earlier for compounds **1a**–**k**, **2a**–**d**, and **2f**–**i** in [54], and for **2e**—in [47].

#### In Silico Analysis

The molecular descriptors of the synthesized spiro-fused acenaphthylene-1,2′-cyclopropa[*a*]pyrrolizines and acenaph-thylene-1,2′-bicyclo[3.1.0]hexanes as well as their aceantrylene analogues were determined with the widely used free online software SwissADME (http://www.swissadme.ch/ accessed on 5 February 2025) and analyzed according to Veber’s rule and Lipinski’s rule.

ADMET profiling was estimated in silico using the PreADMET 2.0 online software (https://preadmet.webservice.bmdrc.org/ accessed on 5 February 2025).

### 3.2. Cell Culture and Culturing Conditions

The human cervical carcinoma (HeLa), erythroleukemia (K-562), and murine melanoma (B16) cell lines were obtained from the Bank of Cell Cultures of the Institute of Cytology of the Russian Academy of Sciences. Human melanoma (Sk-mel-2) and osteosarcoma (U2OS) cell lines were obtained from the Bank of Cell Cultures of the Institute of Cytology and Genetics, Siberian Branch of Russian Academy of Sciences. K-562 cells were cultured in RPMI medium (Hyclone, GE Healthcare Life Sciences, Logan, UT, USA) supplemented with fetal bovine serum (FBS, 10% *v*/*v*, Hyclone, GE Healthcare Life Sciences, Logan, UT, USA) and gentamicin (50 μg/mL, Sigma-Aldrich, St. Louis, MO, USA). Cells of other cell lines (HeLa, Sk-mel-2, U2OS and B16) were cultured in Dulbecco’s Modified Eagle’s Medium (DMEM) medium (Hyclone, GE Healthcare Life Sciences, Logan, UT, USA) with the same supplements. The different cell lines were maintained under controlled conditions: a humid atmosphere with 5% CO_2_ at 37 °C.

### 3.3. Cell Proliferation Assay

Cell viability was measured in vitro using the 3-(4,5-dimethylthiazol-2-yl)-5-(3-carboxymethoxyphenyl)-2-(4-sulfophenyl)-2*H*-tetrazolium (MTS) assay. In short, cells were seeded into 96-well microtiter plates at a density of 5 × 10^3^ cells per well in 100 μL of complete medium and allowed to grow and adhere onto the wells for 24 h at 37 °C. After that, the cells were treated with various concentrations of the compounds for a period of 1 and 3 days. After the treatment, 20 μL of MTS reagent (BioVision, Milpitas, CA, USA) stock solution was added into each well and incubated at 37 °C for 2 h in a humidified, 5% CO_2_ atmosphere. Finally, the absorbance was recorded at 495 nm using 96-well plate reader ‘Multiskan GO’ (Thermo Fisher Scientific, Waltham, MA, USA). All samples were measured in triplicate.

### 3.4. Actin Cytoskeleton Staining

HeLa, Sk-mel-2, or B16 cells were seeded onto a Petri dish with cover slips at a density of 2 × 10^5^ cells per dish and incubated for 24 h. After that, cells were treated with chosen compounds (10 μg/mL) for 24 h. The medium was removed, cells were fixed with 4% paraformaldehyde (Sigma-Aldrich, St. Louis, MO, USA), washed with PBS three times, and permeabilized with 0.3% Triton-X100 (Sigma-Aldrich, St. Louis, MO, USA). The cells were rinsed with PBS three times. Actin filaments (microfilaments) were stained at 37 °C for 15 min with rhodamine-phalloidin (Invitrogen, Carlsbad, CA, USA). The samples were rinsed with PBS three times, followed by embedding in Fluoroshield medium (Sigma-Aldrich, St. Louis, MO, USA). The intensity of the staining of preparations was estimated using an AxioObserver Z1 confocal microscope (Carl Zeiss MicroImaging GmbH, Jena, Germany). In each experiment, at least 30 cells were imaged. Images were processed using ImageJ software 1.54g.

### 3.5. Evaluation of Cell Motility by Scratch Test

Cells were seeded onto Petri dishes at a density of 5 × 10^5^ cells per dish and grown to confluency. Scratch wounds were made by a 200 μL pipette tip and detached cells were removed after that by washing with PBS. Culture media was replaced with serum-free DMEM in order to inhibit cell proliferation. Compounds to be screened were added to the cultures at a 10 μg/mL concentration and incubated for 36 h. Different fields were analyzed by a bright field, and each scratch area was photographed at 0 and 36 h. Images were captured using an Axio Observer Z1 confocal microscope (Carl Zeiss MicroImaging GmbH, Jena, Germany). The percent of wound closure in five randomly chosen fields was calculated with NIH ImageJ software.

### 3.6. Investigation of the Mitochondrial Membrane Potential (∆Ψm) Changes Under Incubation Conditions with Synthesized Compounds

The JC-1 (5,5′,6,6′-tetrachloro-1,1′,3,3′tetraethylbenzimidazolylcarbocyanine chloride; Biotium, Fremont, CA, USA) dye was used as a probe to measure changes in mitochondrial membrane potential in K562 cells treated with the studied compounds. K562 cells were incubated in 24-well microplates at 50,000 cells/well for 24 h. After that, cells were treated with chosen compounds at a concentration of 10 μg/mL. After incubation for 24 h, RPMI was replaced with PBS medium with JC-1 at the final assay concentration of 2 µg/mL; the cells were stained for 20 min at 37 °C and 5% CO_2_ and then washed with PBS. JC-1 exists as a monomer at low concentrations and yields green fluorescence (emission at 530 nm), similar to fluorescein. At higher concentrations or higher mitochondrial potential, JC-1 forms aggregates that exhibit a broad excitation spectrum and an emission maximum at 590 nm. Red and green fluorescence was measured using a standard flow cytometer (BD FACSCanto, Becton Dickinson, San Jose, CA, USA). Results were expressed as the ratio of green to red fluorescence (530/590 nm).

### 3.7. Molecular Docking

Molegro Virtual Docker 6.0 software was used to perform molecular docking. The crystal structure data were obtained from the Protein Data Bank (8DNH, 2Q1N, 7BJ6, and 7BIR). The target structures were prepared automatically using standard procedures of the Molegro Virtual Docker package. The chemical structures of the ligands were drawn using ChemBioDraw Ultra 13.0 and optimized by MM2 calculations in Chem3D Pro 13.0. The MolDock Score was used as a scoring function. There were 20 trial runs for calculations. MolDock SE was used as a docking algorithm following energy minimization and optimization of hydrogen bonds.

### 3.8. Statistical Analysis

Statistical analysis was performed using Statistica 6.0. All data from the three independent experiments were used for measuring the means ± standard deviation (mean ± SD), which were compared using the Student’s *t*-test.

## 4. Conclusions

A series of 3-azabicyclo[3.1.0]hexanes and cyclopropa[*a*]pyrrolizidines spiro-fused to acenaphthylene-1(2H)-one and aceanthrylene-1(2H)-one frameworks have been studied for their in vitro antiproliferative activity against human erythroleukemia (K562), cervical carcinoma (HeLa), melanoma (Sk-mel-2), osteosarcoma (U2OS), as well as murine melanoma (B16) cell lines. It was found that [cyclopropa[a]pyrrolizidines spiro-fused to acenaphthylene-1(2*H*)-one and aceanthrylene-1(2*H*)-one frameworks usually show better antiproliferative activity as compared to 3-azabicyclo[3.1.0]hexanes spiro-fused to acenaph-thylen-1(2*H*)-one and aceanthrylene-1(2*H*)-one frameworks. The adducts spiro-fused to the acenaphthylene-1(2*H*)-one framework are usually more active compared to those with the aceanthrylene-1(2*H*)-one framework. K562 and B16 cell lines were the most sensitive to the screened spiro-fused products among the tested cancer cell lines, with IC_50_ values ranging from 2 ± 1 to 11 ± 5 μg/mL (72 h), which equals to 4–20 μM. Among spiro-fused [cyclopropa[*a*]pyrrolizidines, adduct **1f** with a methoxycarbonyl-substituted cyclopropane ring shows a better antiproliferative effect, with an IC_50_ value of 2 ± 1 μg/mL (which equals to 4 μM) against the K562 cell line, while ethyl (**1b**), cyano (**1g**), and phenyl (**1h**)-substituted spiro-adducts show an antiproliferative effect, with an IC_50_ value of around 6 ± 2 μg/mL (which equals to 12–17 μM). Among the spiro-fused 3-azabicyclo[3.1.0]hexanes, only adducts **2b** and **2c** exhibited a significant antiproliferative effect, with a half maximal inhibitory concentration (IC_50_) of around 14 ± 4 μg/mL (against K562 cell line), which equals to 25–27 μM.

In agreement with the confocal microscopy studies, cultivation with the tested spiro-fused compounds led to the disappearance of stress fibers (granular actin was distributed diffusely in the cytoplasm in up to 56% of treated cells) and decrease in filopodia-like deformations (up to 69% after cultivation), which indirectly suggests a decrease in cell motility. The human melanoma cell line scratch test showed that these cells lose their ability to move after cultivation with the tested spiro-fused compounds and do not fill the scratched strip. This was also supported by docking simulations with actin-related targets (PDB ID: 8DNH, 2Q1N). Using flow cytometry, the impact on the mitochondrial membrane potential showed that tested compounds led to a significant increase in the number of cells with a decreased mitochondrial membrane potential, from 10% for the control up to 55–80% for cyclopropa[*a*]pyrrolizidine adducts and up to 35–75% for azabicyclo[3.1.0]hexane adducts. The obtained results support the antitumor effect of the tested spiro-compounds and encourage the extension of the study in order to improve their anticancer activity as well as reduce their toxicological risks.

## Figures and Tables

**Table 1:** Physicochemical profiles of compounds according to Veber’s and Lipinski’s rule.

Compound	MW	nHBD	nHBA	Log P	nRotB	TPSA, Å^2^	N_Violation_		Meet Lipinski Criteria	Meet Veber Criteria
							Lipinski	Veber		
	<500	<5	<10	≤5	<10	<140	<1	0	Yes/No	Yes/No
**1c**	453.57	0	2	5.54	3	20.31	1	0	Yes	Yes
**1e**	503.63	0	2	6.13	3	20.31	2	0	No	Yes
**1f**	485.57	0	4	4.62	4	46.61	1	0	Yes	Yes
**1h**	519.63	1	3	5.25	3	40.54	2	0	No	Yes
**1i**	521.67	0	2	5.95	3	45.61	2	0	No	Yes
**2b**	519.67	1	2	6.30	6	29.10	2	0	No	Yes
**2c**	519.67	1	2	6.30	5	29.10	2	0	No	Yes
**2d**	537.71	1	2	6.13	6	54.40	2	0	No	Yes
**2f**	569.73	1	2	6.82	6	29.10	2	0	No	Yes

MW: molecular weight; nHBD: number of hydrogen-bond donors; nHBA: number of hydrogen bond acceptors; Log P: logarithm of partition coefficient of the compound between n-octanol and water; nRotB: number of rotatable bonds; TPSA: topological polar surface area; N_Violation_: number of violated criteria.

**Table 2:** ADMET profiles of selected compounds.

Compound	HIA, %	BBB	PPB, %	CYP_2D6 Inhibition	Solubility, mg/L	Carcinogenicity (Rat/Mouse)	Mutagenicity	hERG Inhibition
**1c**	100	2.01	93,41	inhibitor	0.0036	negative/negative	mutagen	low risk
**1e**	100	2.53	97.22	inhibitor	0.0005	negative/negative	non-mutagen	low risk
**1f**	98.17	5.69	90.55	inhibitor	0.0224	negative/negative	mutagen	low risk
**1h**	96.97	1.59	100	inhibitor	0.0010	negative/negative	mutagen	low risk
**1i**	98.31	5.44	100	inhibitor	9 × 10^−5^	negative/negative	non-mutagen	low risk
**2b**	97.18	10.22	100	inhibitor	4 × 10^−5^	positive/negative	non-mutagen	low risk
**2c**	97.18	5.89	100	inhibitor	5 × 10^−5^	negative/negative	mutagen	low risk
**2d**	97.64	0.36	100	inhibitor	0.0001	negative/negative	non-mutagen	low risk
**2f**	96.85	9.74	100	inhibitor	7 × 10^−7^	positive/negative	non-mutagen	low risk

HIA: human intestinal absorption; BBB: in vivo blood–brain barrier penetration (*C. brain*/*C. blood*); PPB: in vitro plasma protein binding; CYP_2D6_inhibition: in vitro Cytochrome P450 2D6 inhibition; solubility: water solubility in pure water mg/L; carcinogenicity: 2 years carcinogenicity bioassay in rat and mouse; mutagenicity: mutagenicity according to Ames test; hERG inhibition: in vitro human ether-a-go-go related gene channel inhibition.

**Table 3:** IC_50_ values of most active spiro-fused products against **K562**, **U2OS**, and **B16** cell lines for 72 h.

Compound	IC_50_, μM			Compound	IC_50_, μM		
	K562	U2OS	B16		K562	U2OS	B16
**1a**	>40	>40	15 ± 3	**1h**	12 ± 2	28 ± 7	4 ± 1
**1b**	13 ± 2	>40	10 ± 2	**1i**	>40	>40	13 ± 4
**1c**	>40	>40	11 ± 2	**1j**	>40	>40	20 ± 5
**1d**	>40	>40	15 ± 4	**2b**	27 ± 5	>40	22 ± 7
**1e**	>40	>40	7 ± 2	**2c**	25 ± 4	>40	>40
**1f**	4 ± 1	>40	7 ± 2	Doxorubicin	1 ± 0.3	7 ± 2	6 ± 1
**1g**	17 ± 4	>40	8 ± 2				

**Table 4:** Docked study results of **1b**, **1c**, **1e**, **1f**, **1g**, **1i**, **2b**, **2c**, and **2d** with the target proteins (PDB ID: 8DNH).

#	Protein PDB ID	Ligand	MolDock Score ^a^	Rerank Score ^a^	HBond ^a^	Amino Acid Residue ID (Target-Binding Cleft) ^b^
1	8DNH	**1b**	−153.95	−97.868	0	Ala 107, Arg 115, Asn 110, Cys 373, Glu 106, His 172, His 370, Ile 135, Ile 174, Leu 109, Lys 112, Phe 374, Pro 108, Pro 111, Pro 171, Val 369, Glu 194, Lys 190, Thr 193
2	8DNH	**1c**	−159.21	−100.86	0	Ala 107, Arg 115, Asn 110, Cys 373, Glu 106, His 172, His 370, Ile 135, Ile 174, Leu 109, Lys 112, Phe 374, Pro 108, Pro 111, Pro 171, Val 369, Glu 194, Lys 190, Thr 193
3	8DNH	**1e**	−160.79	−101.22	0	Arg 115, Arg 371, Asn 110, Glu 106, His 172, His 370, Ile 135, Ile 174, Leu 109, Lys 112, Phe 374, Pro 108, Pro 111, Pro 171, Val 369, Glu 194, Lys 190, Thr 193
4	8DNH	**1f**	−157.07	−99.393	−5.088	Ala 107, Arg 115, Asn 110, Cys 373, Glu 106, His 172, His 370, Ile 135, Ile 174, Leu 109, Lys 112, Phe 374, Pro 108, Pro 111, Pro 171, Val 369, Glu 194, Lys 190, Thr 193
5	8DNH	**1g**	−152.95	−98.533	−1.274	Ala 107, Arg 115, Asn 110, Glu 106, His 172, His 370, Ile 135, Ile 174, Leu 109, Lys 112, Phe 374, Pro 108, Pro 111, Pro 171, Val 369, Glu 194, Lys 190, Thr 193
6	8DNH	**1i**	−163.94	−99.137	0	Ala 107, Arg 115, Arg 371, Asn 110, Glu 106, His 172, His 370, Ile 135, Ile 174, Leu 109, Lys 112, Phe 374, Pro 108, Pro 111, Pro 171, Val 369, Glu 194, Lys 190, Thr 193
7	8DNH	**2b**	−177.53	−105.71	0	Ala 107, Arg 115, Arg 371, Asn 110, Cys 373, Glu 106, His 172, His 370, Ile 135, Ile 174, Leu 109, Lys 112, Phe 374, Pro 108, Pro 111, Pro 171, Val 133, Val 369, Glu 194, Lys 190, Thr 193
8	8DNH	**2c**	−172.68	−93.067	−2.874	Ala 107, Ala 134, Ala 169, Arg 115, Asn 110, Cys 373, Glu 106, His 370, Ile 135, Ile 174, Leu 109, Leu 170, Lys 112, Phe 374, Pro 108, Pro 111, Pro 171, Tyr 132, Val 133, Val 138, Val 162, Val 42
9	8DNH	**2d**	−176.60	−115.24	−0.799	Ala 107, Ala 134, Ala 169, Arg 115, Asn 110, Cys 373, Glu 106, His 370, Ile 135, Ile 174, Leu 109, Leu 170, Lys 112, Phe 374, Pro 108, Pro 111, Pro 171, Tyr 132, Val 133, Val 138, Val 162, Val 42

^a^ arbitrary units; ^b^ contributed to the interaction with docked ligand amino acids, according to ligand energy inspector.

**Table 5:** Docked study results of **1b**, **1c**, **1e**, **1f**, **1g**, **1i**, **2b**, **2c**, and **2d** with the target proteins (PDB ID: 7BJ6, 7BIR).

#	Protein PDB ID	Ligand	MolDock Score ^a^	Rerank Score ^a^	HBond ^a^
1	7BJ6	TVK*	−159.09	−108.74	−2.261
2	7BJ6	1b	−142.49	−88.908	0
3	7BJ6	1c	−134.28	−83.325	0
4	7BJ6	1e	−138.17	−77.631	0
5	7BJ6	1f	−132.48	−77.205	0
6	7BJ6	1g	−130.21	−76.526	0
7	7BJ6	1i	−135.84	−76.857	0
8	7BJ6	2b	−159.08	−97.295	−0.764
9	7BJ6	2c	−148.45	−74.777	0
10	7BJ6	2d	−158.34	−85.999	0
11	7BJ6	TUZ	−163.89	−111.47	−2.918
12	7BIR	TUZ*	−166.93	−112.48	−3.878
13	7BIR	1b	−139.12	−85.431	0
14	7BIR	1c	−135.18	−81.189	0
15	7BIR	1e	−138.72	−72.773	0
16	7BIR	1f	−134.55	−69.128	0
17	7BIR	1g	−137.06	−82.389	0
18	7BIR	1i	−136.86	−68.419	0
19	7BIR	2b	−158.62	−96.458	0
20	7BIR	2c	−151.68	−81.008	0
21	7BIR	2d	−162.68	−94.685	0
22	7BIR	TVK	−145.61	−96.633	−5.341

^a^ arbitrary units; TVK*, TUZ*—active ligands for 7BJ6 and 7BIR, respectively, TVK—placed to 7BIR for docking study, TUZ—placed to 7BJ6 for docking study.

## Data Availability

The data presented in this study are available on reasonable request from the corresponding authors.

## Supplementary Materials

The following supporting information can be downloaded at: https://www.mdpi.com/article/10.3390/ijms26083474/s1.
