# Supplementary material for: Study of Cytotoxicity of 3-Azabicyclo[3.1.0]hexanes and Cyclopropa[a]pyrrolizidines Spiro-Fused to Acenaphthylene-1(2H)-one and Aceanthrylene-1(2H)-one Fragments Against Tumor Cell Lines
Source: Int J Mol Sci. 2025 Apr 8;26(8):3474. doi: 10.3390/ijms26083474 (PMC12026830; doi:10.3390/ijms26083474)
Supplement: Supplementary file 1 [file ijms-26-03474-s001.zip › ijms-3516187-supplementary.pdf]

International Journal of *Molecular Sciences*

MDPI

Supporting Information

Study of Cytotoxicity of 3-Azabicyclo[3.1.0]hexanes and Cyclopropa[*a*]pyrrolizidines Spiro-Fused to Acenaphthylene-1(2*H*)-one and Aceanthrylene-1(2*H*)-one Fragments Against Tumor Cell Lines

Anton A. Kornev [1], Stanislav V. Shmakov [1], Alexandra M. Gryschenko [1], Yulia A. Pronina [2], Alexander I. Ponyaev [2], Alexander V. Stepakov [2,3,*] and Vitali M. Boitsov [1,*]

[1] Laboratory of Nanobiotechnologies, Saint-Petersburg National Research Academic University of the Russian Academy of Sciences, Saint Petersburg 194021, Russia
[2] Saint-Petersburg State Institute of Technology, Saint Petersburg 190013, Russia
[3] Department of Chemistry, Saint-Petersburg State University, Saint Petersburg 199034, Russia
* Correspondence: bovitali@yandex.ru (V.M.B.); alstepakov@yandex.ru (A.V.S.)

Synthesis of racemic spiro-fused adducts **1a–k** and **2a–i**

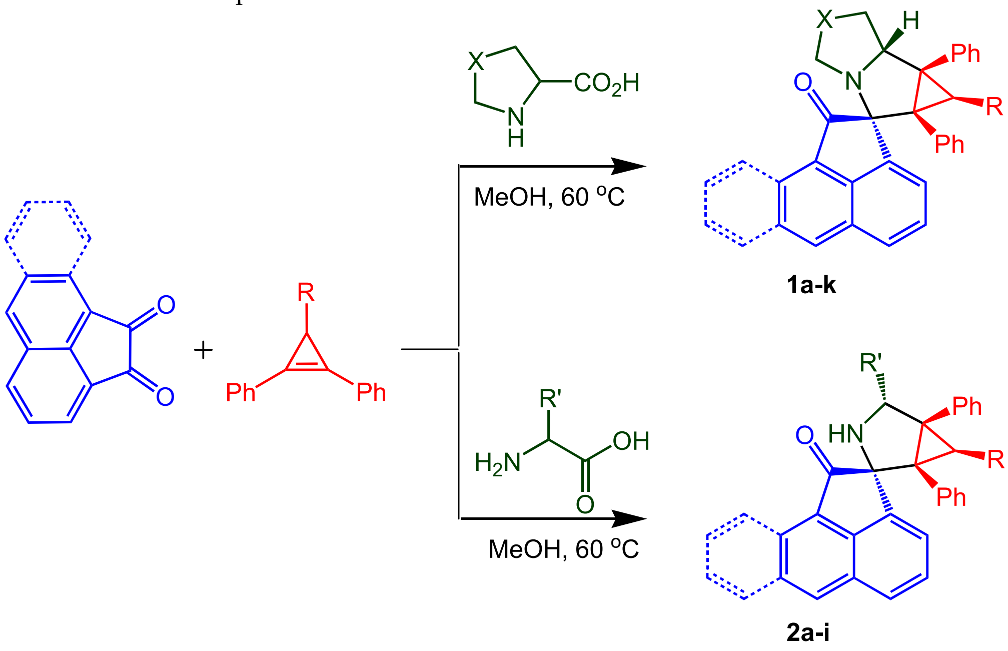

Scheme S1. Synthesis of racemic spiro-adducts **1a–k** and **2a–i.**

Antiproliferative activity study of synthesized spiro-adducts against human erythroleukemia (K562), cervical carcinoma (HeLa), melanoma (Sk-mel-2) and osteosarcoma (U2OS) as well as murine melanoma (B16) cell lines.

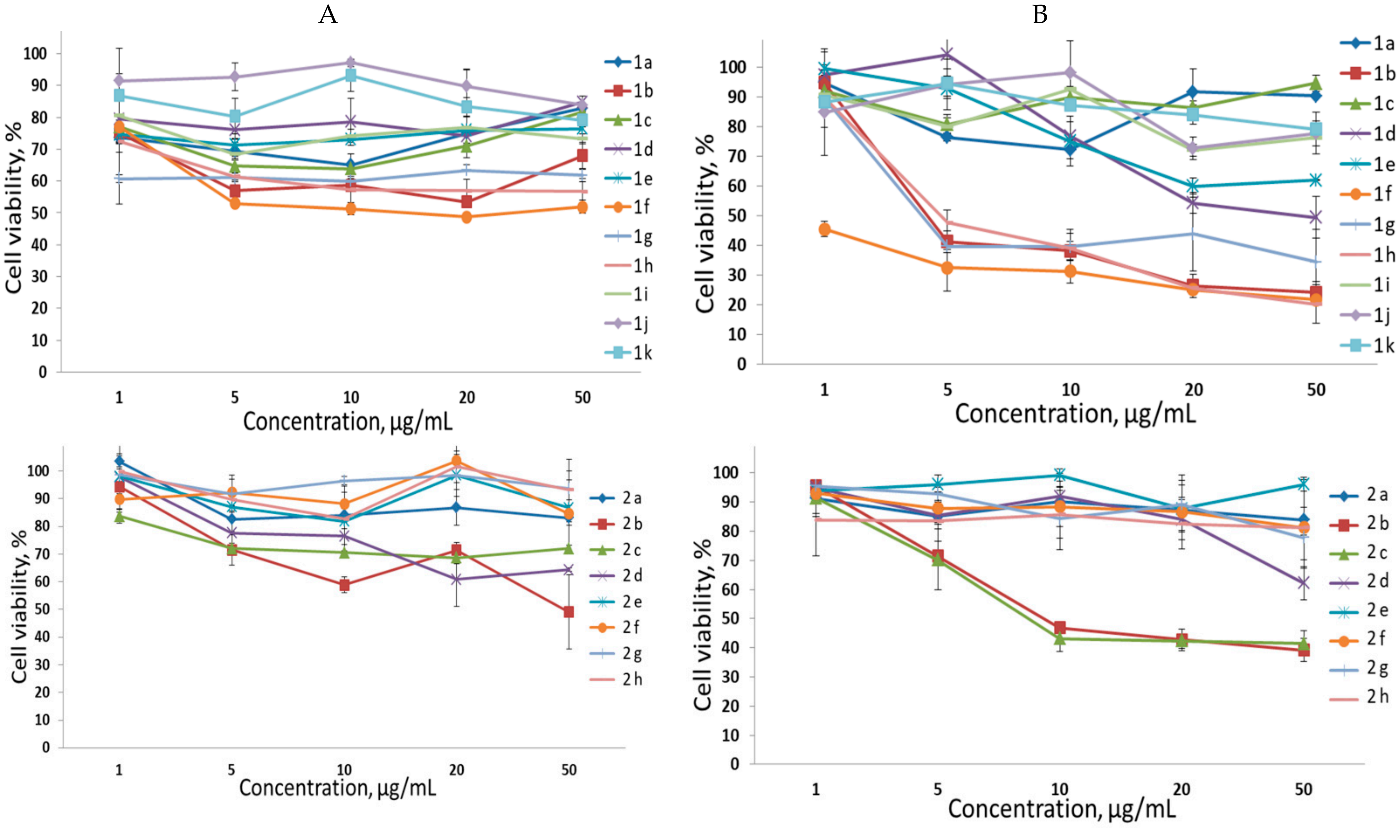

Figure S1. Cytotoxicity of racemic spiro-fused cyclopropa[a]pyrrolizines **1a–k** and 3-azabicyclo[3.1.0]hexanes **2a–h** against the K562 cell line for 24 h (**A**) and 72 h (**B**).

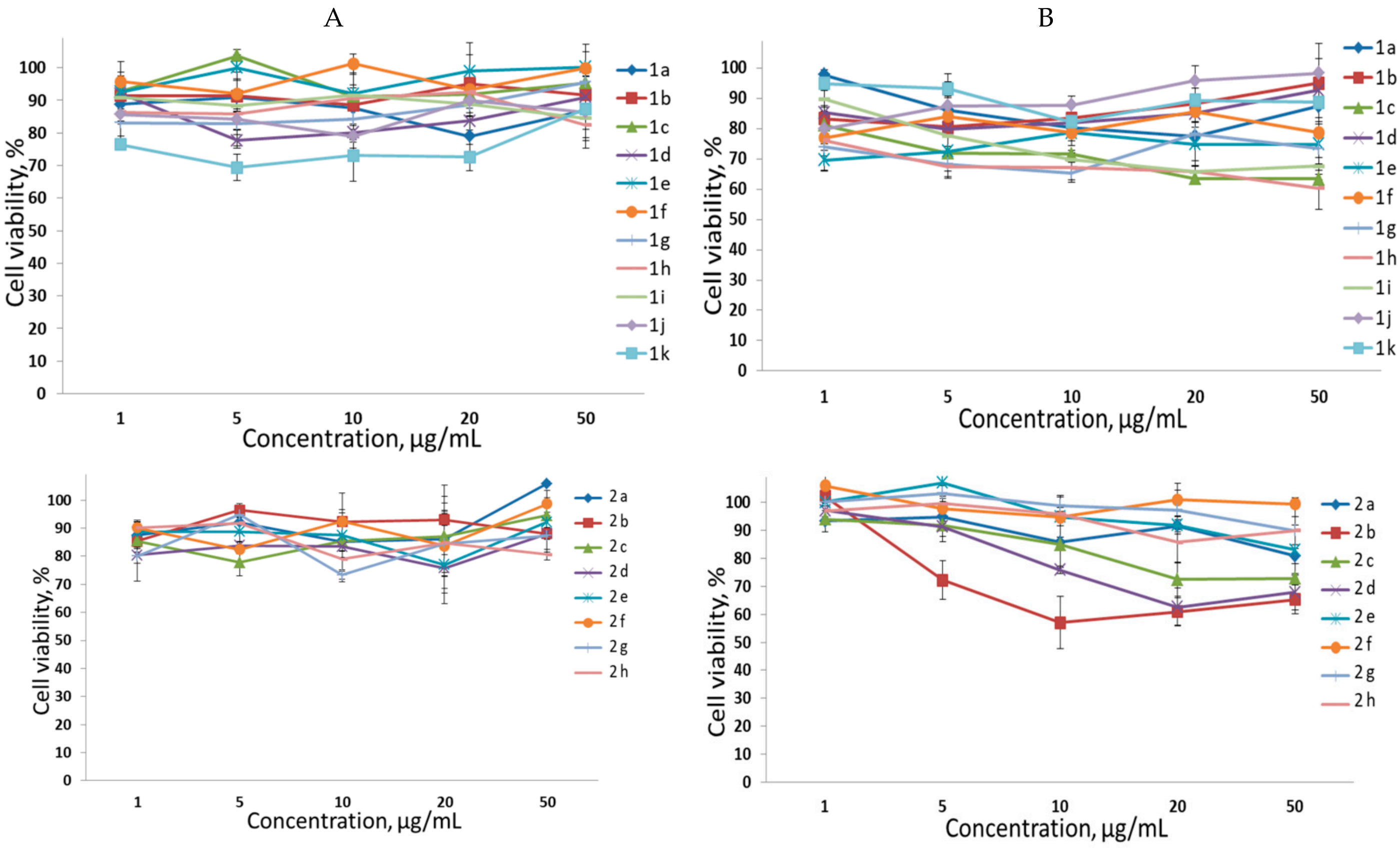

Figure S2. Cytotoxicity of racemic spiro-fused cyclopropa[a]pyrrolizines **1a–k** and 3-azabicyclo[3.1.0]hexanes **2a–h** against the HeLa cell line for 24 h (**A**) and 72 h (**B**).

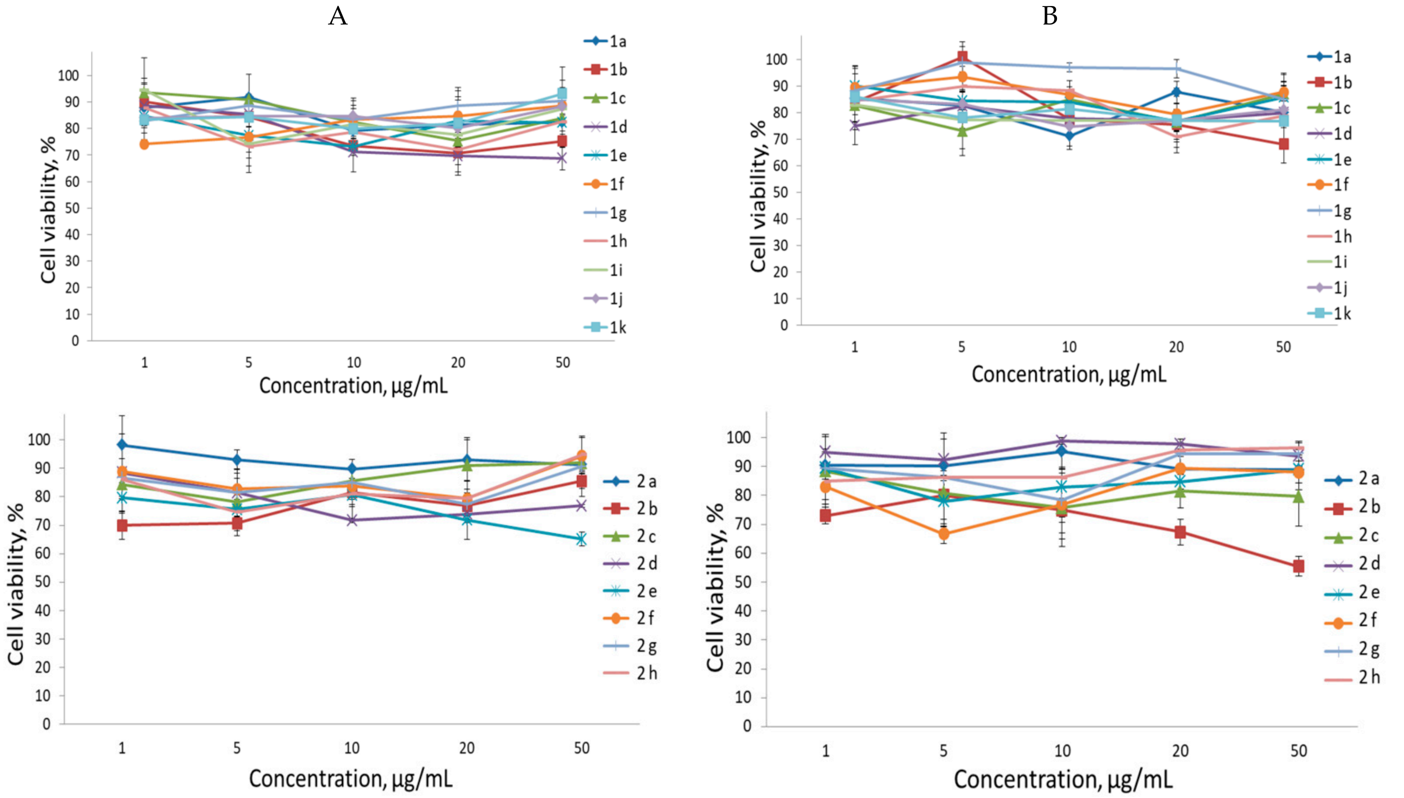

Figure S3. Cytotoxicity of selected racemic spiro-fused cyclopropa[a]pyrrolizines **1a–k** and 3-azabicyclo[3.1.0]hexanes **2a–h** against the Sk-mel-2 cell line for 24 h (**A**) and 72 h (**B**).

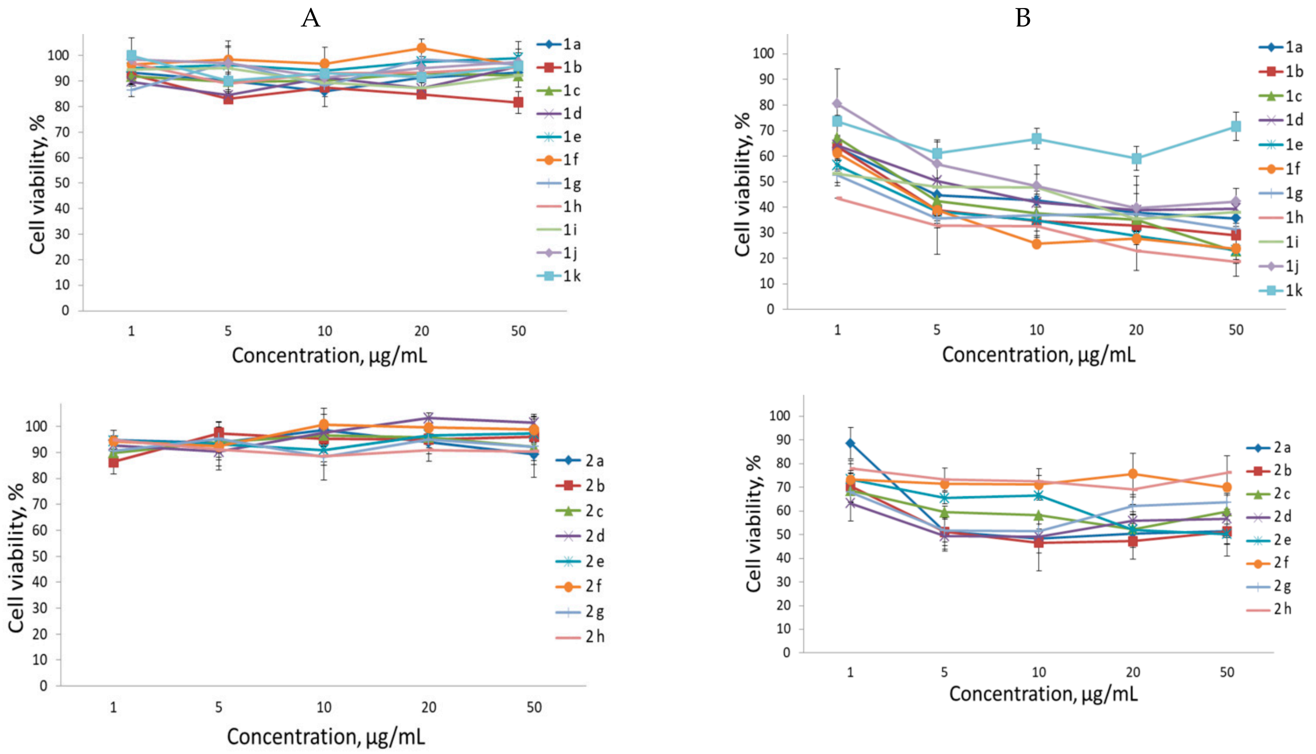

Figure S4. Cytotoxicity of selected racemic spiro-fused cyclopropa[a]pyrrolizines **1a–k** and 3-azabicyclo[3.1.0]hexanes **2a–h** against the B16 cell line for 24 h (**A**) and 72 h (**B**).

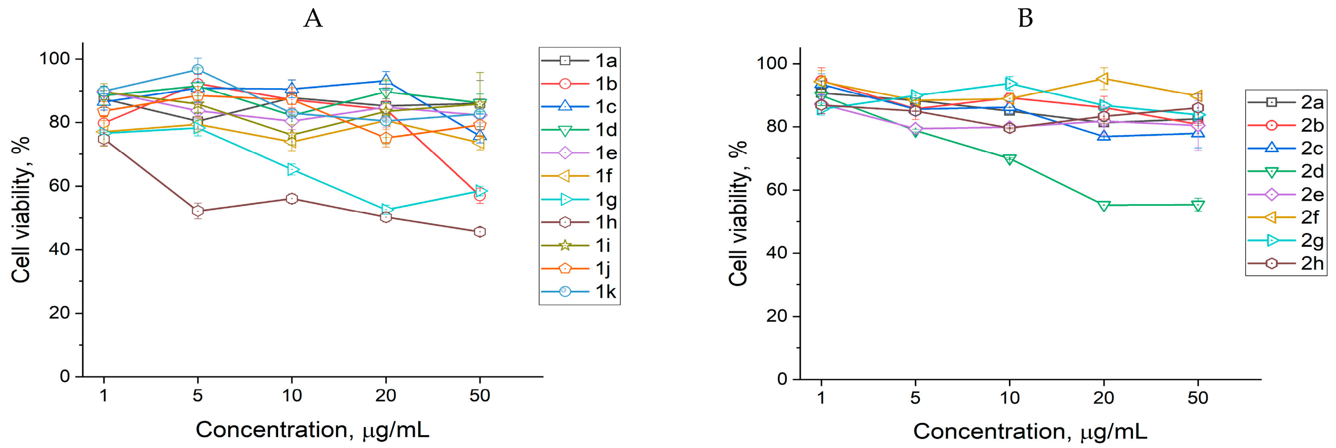

Figure S5. Cytotoxicity of racemic spiro-fused cyclopropa[*a*]pyrrolizines **1a–k** (A) and 3-azabicyclo[3.1.0]hexanes **2a–i** (B) against the U2OS cell line for 72 h..

Molecular docking simulations

Table S1. Docked study results of **1b, 1c, 1e, 1f, 1g, 1i, 2b, 2c** and **2d** with the target proteins.

#	**Protein PDB ID**	**Ligand**	**MolDock Score** [a]	**Rerank Score** [a]	**HBond** [a]	**MolDock Score** [a]	**Rerank Score** [a]	**HBond** [a]
			nucleotide cleft			target-binding cleft		
1	8DNH	1b	-131.73	-66.859	0	-153.95	-97.868	0
2	8DNH	1c	-126.52	-59.685	0	-159.21	-100.86	0
3	8DNH	1e	-165.90	-99.005	0	-160.79	-101.22	0
4	8DNH	1f	-126.91	-62.199	0	-157.07	-99.393	-5.088
5	8DNH	1g	-130.54	-59.534	-2.211	-152.95	-98.533	-1.274
6	8DNH	1i	-131.43	-79.472	0	-163.94	-99.137	0
7	8DNH	2b	-148.08	-22.749	-1.973	-177.53	-105.71	0
8	8DNH	2c	-170.30	-86.694	-2.489	-172.68	-93.067	-2.874
9	8DNH	2d	-146.78	-72.027	-0.588	-176.60	-115.24	-0.799
10	8DNH	ADP	-166.99	-136.70	-12.06	-132.39	-112.54	-14.42
11	2Q1N	1b	-124.18	-74.510	0	-138.98	-86.396	0
12	2Q1N	1c	-124.08	20.726	2.885	-147.35	-96.658	0
13	2Q1N	1e	-136.32	-71.812	-1.469	-153.57	-99.551	0
14	2Q1N	1f	-146.43	-93.076	-0.226	-146.42	-93.076	-0.2261
15	2Q1N	1g	-121.88	-70.115	0	-141.65	-92.900	-0.5313
16	2Q1N	1i	-137.37	-74.815	-1.538	-151.48	-96.915	0
17	2Q1N	2b	-158.21	-63.842	-0.8057	-154.05	-97.622	-2.500
18	2Q1N	2c	-152.76	-88.916	-0.4593	-147.66	-89.746	-2.033
19	2Q1N	2d	-154.91	-56.686	-0.477522	-150.17	-92.770	-0.541
20	2Q1N	ANP	-229.69	-181.20	-22.32	-153.11	-114.53	-13.24

[a] arbitrary units; ADP – Adenosine diphosphate, ANP – Adenylyl imidodiphosphate

8DNH

2Q1N

A

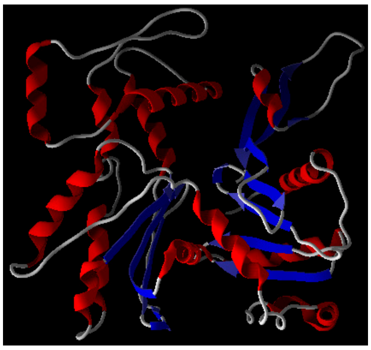

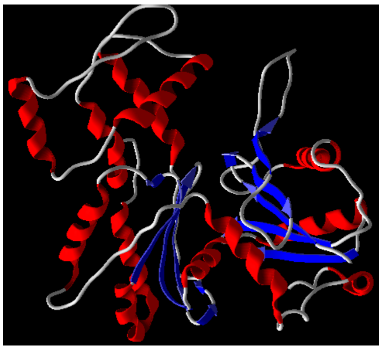

B

1c

1f

2b

2c

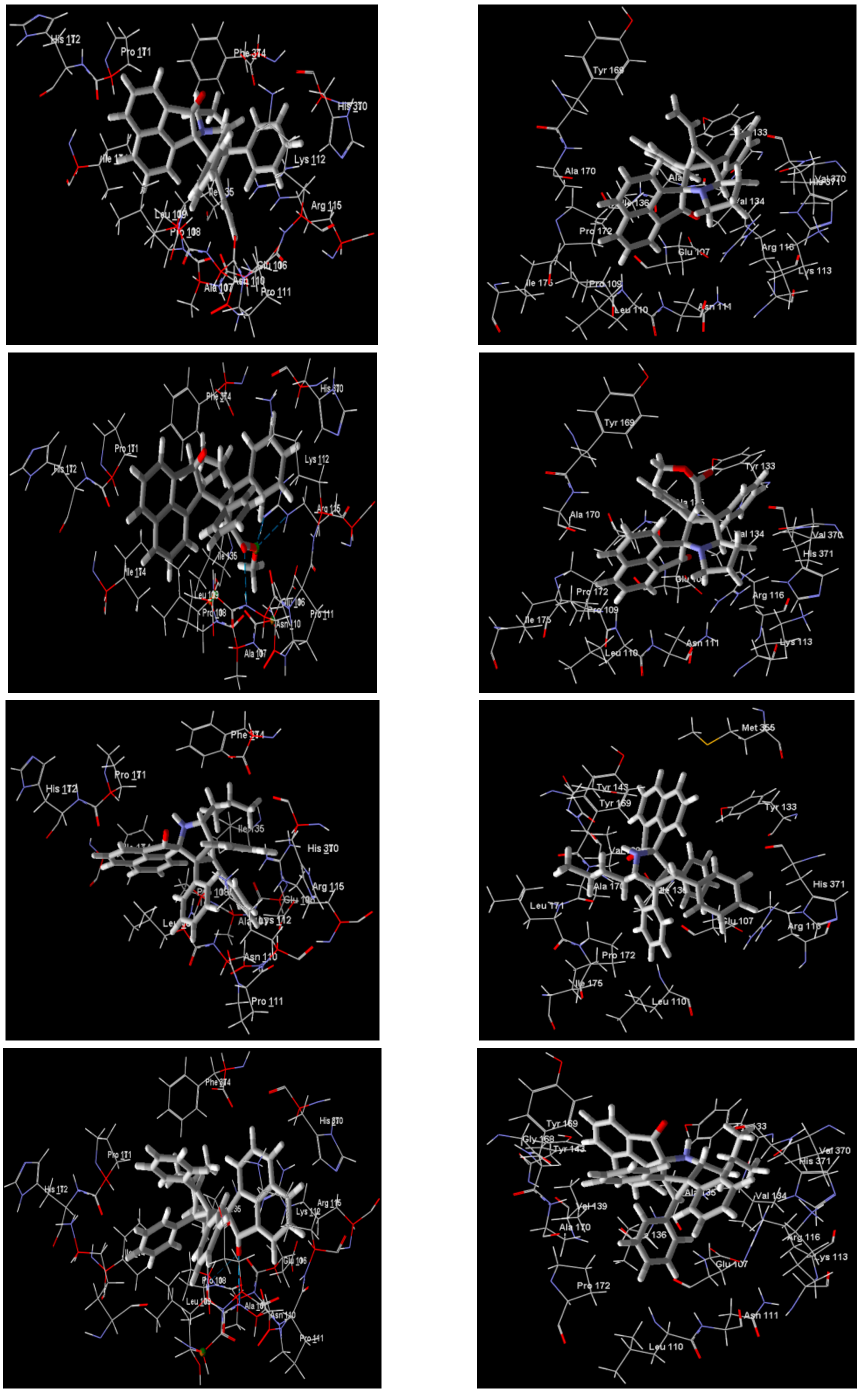

Figure S6. Predicted binding models of compounds **1c, 1f, 2b** and **2c** within the target-binding cleft of actin proteins. A: Classical view of the structure of the actin monomers, B: structures of actin complexes (PDB ID: 8DNH, 2Q1N) with small molecules.

7BIR 7BJ6

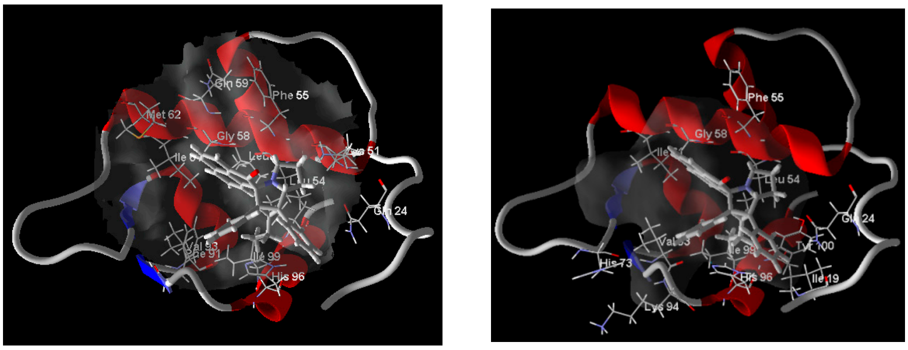

1b

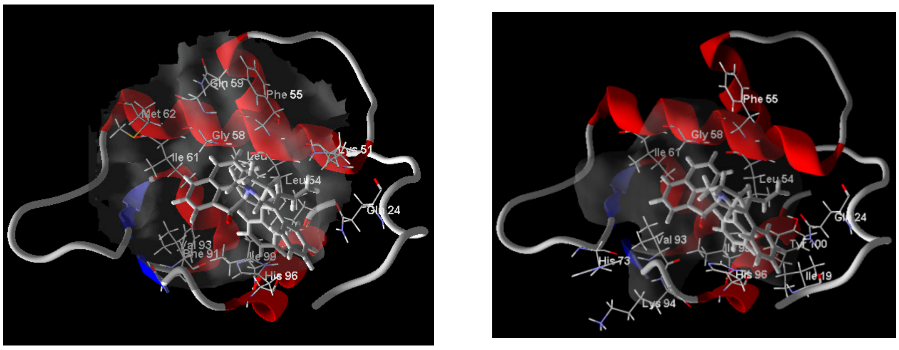

1c

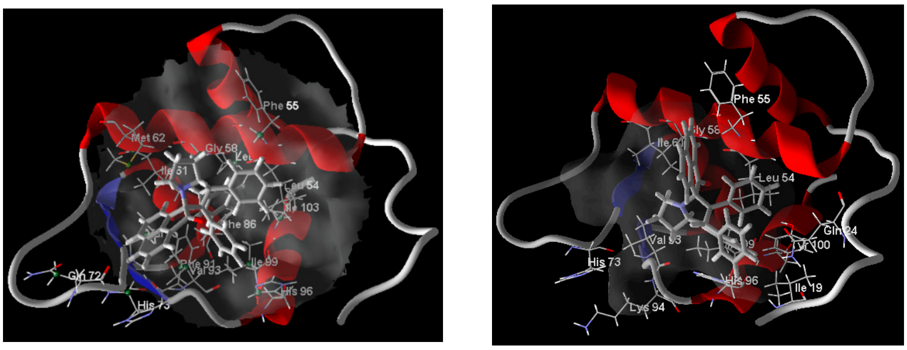

1e

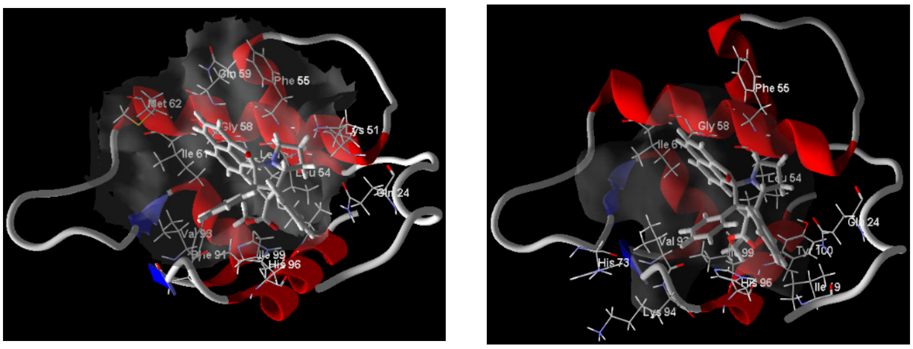

1f

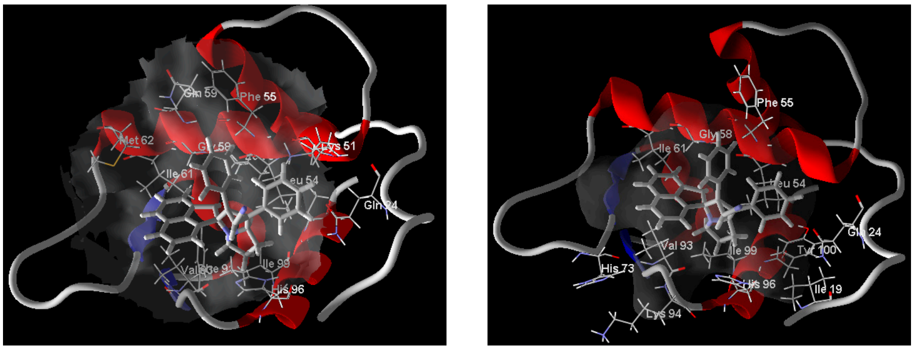

1g

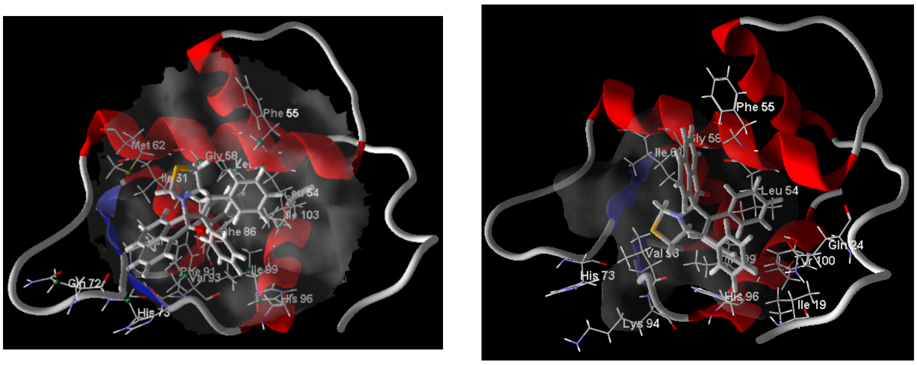

1i

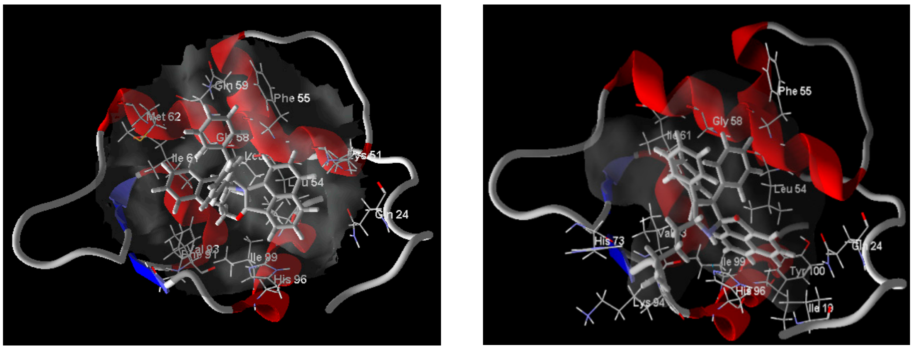

2b

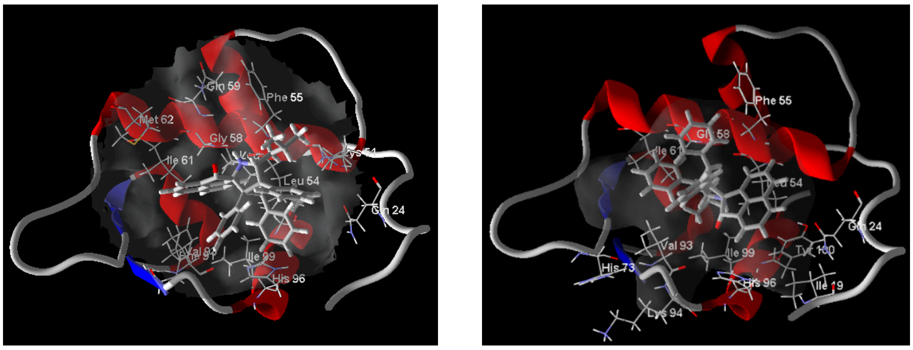

2c

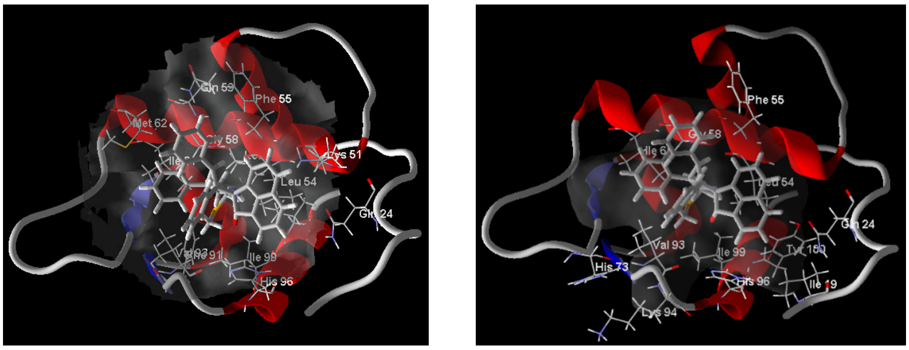

2d

Figure S7. Predicted binding models of compounds **1b, 1c, 1e, 1f, 1g, 1i, 2b, 2c** and **2d** within the target-binding cleft of MDM2 protein (for PDB ID: 7BIR and 7BJ6).
